# *GmAGL6* Genes Regulate Floral Proportion and Seed Size Rather than Keel Petal Identity in Soybean (*Glycine max*)

**DOI:** 10.3390/plants15071070

**Published:** 2026-03-31

**Authors:** Haoming Zhai, Yezhou Liu, Meng Xia, Liwen Tang, Siyuan Zheng, Liangsheng Zhang, Dan Chen

**Affiliations:** 1 Hainan Institute, Zhejiang University, Sanya 572025, China; 22316172@zju.edu.cn (H.Z.); 22316170@zju.edu.cn (Y.L.); summer0501xm@outlook.com (M.X.); 22316216@zju.edu.cn (L.T.); 22216132@zju.edu.cn (S.Z.); 2 Zhejiang Provincial Key Laboratory of Horticultural Plant Integrative Biology, College of Agriculture and Biotechnology, Zhejiang University, Hangzhou 310058, China; 3 Yazhouwan National Laboratory, Sanya 572025, China

**Keywords:** soybean (*Glycine max*), *AGL6* gene family, CRISPR/Cas9, floral organ development, seed size, functional divergence, MADS-box

## Abstract

*AGL6* genes are critical floral regulators in diverse angiosperms, yet their roles in legumes remain poorly understood. This study aimed to characterize *GmAGL6* genes in soybean (*Glycine max* [L.] Merr. cv. Williams 82). We identified four homologs (*GmAGL6a–d*) featuring conserved MADS-box and K-box domains that cluster within the *AGL6* lineage. Tissue-specific expression profiling revealed significant transcript enrichment during flower bud differentiation and maturation. Using CRISPR/Cas9, we generated quadruple knockout lines to evaluate gene function. Phenotypic analysis showed that, unlike the homeotic transformations typical of *AGL6* loss in monocots, *Gmagl6* quadruple mutants retained a standard papilionaceous floral structure without keel petal aberrations. However, the mutants did not show significant changes in floral height or width, but exhibited a significantly increased floral height-to-width ratio and smaller mature seeds, while vegetative architecture and podding capacity remained unaffected. These results suggest that *GmAGL6* genes in soybean may function primarily in the regulation of floral proportion and seed development rather than floral organ identity. This research provides insights into the evolution of specialized legume flowers and suggests candidate genes for seed size improvement.

## 1. Introduction

The staggering diversity of floral morphology represents a hallmark of angiosperm evolution. Central to our understanding of this process is the ABCDE model of floral organogenesis, which posits that the formation and identity of floral organs are orchestrated by MADS-box transcription factors—specifically the plant-specific, classical MIKC^c^-type—functioning as pivotal regulators across multiple developmental stages [1,2,3]. Research in the model organism *Arabidopsis thaliana* has established that A-, B-, C-, D-, and E-class genes assemble into specific transcriptional complexes to dictate the identities of sepals, petals, stamens, and carpels [4]. Within this framework, B-class genes (e.g., *PI* and *AP3*) primarily control petal and stamen development; the C-class gene *AGAMOUS* (*AG*) works in tandem with B-class genes to specify stamens and independently governs carpel formation, while simultaneously antagonizing A-class gene expression to define organ boundaries [5]. The *AG* gene and its *AGAMOUS-Like* (*AGL*) relatives comprise essential components of the C-, D-, and E-class families [6,7,8,9,10]. Current evidence highlights the *AGAMOUS-Like 6* (*AGL6*) family as pivotal for floral meristem differentiation, organ specification, and ovule development across diverse angiosperms [11,12,13,14].

Beyond *Arabidopsis*, investigations in various dicot species have further underscored the role of *AGL6* in organ identity. In *Petunia hybrida*, analysis of *phagl6* and *fbp2* mutants demonstrated that *PhAGL6* functions redundantly with *AGL2* to drive petal and stamen development; their dual loss causes petals to revert to green sepaloid structures, emphasizing a tight functional link between these genes [12,15,16]. Similarly, silencing the tomato (*Solanum lycopersicum*) *SlAGL6* gene leads to sepal fusion and virescent petals by disrupting the expression of A-class (*MC*) and B-class (*TM6*) genes [17]. More recently, *SlAGL6* has been identified as a key inhibitor of fertilization-independent fruit set (parthenocarpy) in tomato. It acts within the ovule integument to suppress cell proliferation regulators like *SlKLUH*; its loss-of-function leads to enlarged ovules due to integument over-proliferation, uncoupling fruit development from fertilization [18]. In the Mesangiosperms *Chimonanthus praecox*, *CpAGL6* participates in organ identity determination, showing high expression levels in middle and inner tepals [19]. Overexpression of *CpAGL6* in *Arabidopsis* results in carpel abnormalities, stunted vegetative growth, and accelerated flowering—the latter driven by the downregulation of the floral repressor FLC and the upregulation of promoters *AP1* and *FT* [20]. Similarly, the orchid *AGL6* homolog *OMADS1* acts as a potent floral activator; its ectopic expression in heterologous systems promotes early flowering and causes a marked reduction in plant dimensions, reinforcing the conserved function of the *AGL6* lineage in orchestrating the transition to flowering and modulating overall plant architecture [21]. In another basal angiosperm, *Nymphaea caerulea*, *AGL6* acts as an A-class gene predominantly expressed in petals and sepals, whereas *FUL-like* genes are mainly confined to the carpel [22].

In monocots, *AGL6* orthologs are indispensable for specifying the identities of paleae, lodicules, stamens, and pistils; mutations in these genes typically trigger severe homeotic transformations or malformations [23]. In rice (*Oryza sativa*), the *OsMADS6* ortholog is essential for palea identity; *osmads6* mutants exhibit paleae transformed into lemma-like structures and lack the marginal reproductive area (MRP) [10,24,25,26]. These mutants also display lodicule and stamen defects, as well as pistil abnormalities such as supernumerary stigmas and fused carpels [10,24,25,26]. Similarly, the maize (*Zea mays*) *AGL6* homolog *ZAG3* regulates floral meristem fate, with mutants showing excessive proliferation and the development of ectopic organs [27]. In wheat (*Triticum aestivum*), *TaAGL6* deficiency leads to the infinite proliferation of spikelet-like structures instead of pistils, indicating a complete loss of meristem determinacy [28]; recent findings further confirm *AGL6* as a master regulator of palea morphogenesis in wheat [29]. In barley (*Hordeum vulgare*), *hvagl6* mutations cause paleae, lodicules, and stamens to transform into lemma-like organs, resulting in total sterility [30].

Recent comparative functional analyses have revealed that the *AP1/SEP/AGL6* superclade exhibits highly divergent functional diversification patterns across angiosperms. For instance, while *AGL6* in petunia functions redundantly with *SEP* genes to provide essential E-class functions, such roles appear less prominent or specialized in other lineages, suggesting that the functional trajectory of the *AGL6* lineage is contingent upon species-specific regulatory networks [16].

Orchids represent one of the most morphologically specialized monocot lineages, possessing a unique floral architecture [31,32,33]. A defining feature is the labellum (lip), a specialized petal essential for attracting specific pollinators and driving diversification. Based on genomic studies of *Phalaenopsis*, a ‘P-code’ model was proposed to explain perianth development [34,35,36]. This model posits that distinct B-class and AGL6-like proteins form two heterotetrameric complexes: the SP complex (*OAP3-1/OAGL6-1/OPI*) controls sepal and petal development, while the L complex (*OAP3-2/OAGL6-2/OPI*) specifically governs labellum formation. Silencing *OAGL6-2* causes the labellum to lose its identity and revert to a sepal/petal-like structure, with its characteristic conical epidermal cells becoming flattened. More broadly, recent work in *Phalaenopsis* has shown that floral trait formation in orchids is governed by a complex regulatory network involving flowering-time pathways, MADS-box genes, floral symmetry regulators, and pigmentation- and size-related factors, highlighting the developmental complexity of orchid floral specialization [37]. In addition to its role in specialized floral organ development, functional evidence from orchids also suggests a broader regulatory capacity of the *AGL6* lineage. In *Oncidium Gower Ramsey*, ectopic expression of the *AGL6-like* gene *OMADS1* in *Arabidopsis thaliana* promoted early flowering by activating flowering-time genes, indicating that *AGL6* homologs may participate not only in floral organ development but also in the regulation of floral transition [21].

Despite its established importance in other families, the role of *AGL6* in legumes remains poorly understood. In *Medicago sativa*, *AGL6* modulates nodulation-related genes by interacting with *SPL12*, and its silencing helps maintain nodulation under osmotic stress [38]. Regarding floral development, previous work in soybean (*Glycine max*) hypothesized that *GmAGL6* members might collaborate with key regulators like *GmAP1* or *GmFLC* to facilitate developmental plasticity [1]. Legume flowers possess a specialized papilionaceous structure composed of three distinct petal types: the standard, wings, and fused keels [39]. While the keel is vital for pollination mechanisms, its genetic regulation is unclear. Although B-class genes are known to function in soybean floral development [40], the role of E-class genes—specifically *AGL6* homologs—in constructing the papilionaceous flower has not been reported.

In the present study, orchid AGL6 sequences were employed to identify four homologs (*GmAGL6a–d*) in the soybean genome, which were subsequently characterized via phylogenetic, domain, and expression profiling. To investigate their regulatory roles in floral development, CRISPR/Cas9-mediated quadruple mutants were generated. Phenotypic characterization revealed that, unlike the homeotic transformations typical of *AGL6* loss in monocots, the *Gmagl6* quadruple mutants maintained a standard papilionaceous floral architecture with intact keel petals. However, loss of *GmAGL6* function did not alter keel petal identity or significantly change floral height or width, but significantly increased the floral height-to-width ratio and reduced mature seed size. These findings indicate that *GmAGL6* genes have undergone functional divergence in soybean, transitioning from ancestral organ identity determination to the regulation of reproductive organ proportion and seed development.

## 2. Results

### 2.1. Phylogenetic and Structural Characterization of the AGL6 Gene Family

As essential members of the Type II MADS-box transcription factor family, *AGL6* genes are master regulators of floral organ development. Prior studies in orchids (*Cymbidium* ssp.) have demonstrated that the loss-of-function of the *OAGL6-2* gene results in the complete disappearance of the labellum [35,41], highlighting its central role in floral morphogenesis. To elucidate the evolutionary trajectory of this family and determine the phylogenetic status of soybean *AGL6* homologs, we utilized the orchid OAGL6-2 protein sequence as a query to perform homology screening across the genomes of 18 representative species using HMMER v3.3.2 (E-value ≤ 1 × 10^−4^) [42]. The selected species spanned a comprehensive evolutionary lineage from lower photosynthetic organisms to higher angiosperms, including algae (*Chlorella vulgaris*), bryophytes (e.g., *Marchantia polymorpha*), gymnosperms (*Gnetum montanum*, *Ginkgo biloba*), basal angiosperms (*Amborella trichopoda*, *Nymphaea tetragona*, *Nymphaea thermarum*), monocots (*Oryza sativa*, *Zea mays*, *Musa acuminata*), and dicots (*Glycine max*, *Arabidopsis thaliana*, *Solanum lycopersicum*).

Candidate sequences identified via HMMER were initially aligned using MAFFT 7.475 (L-INS-i algorithm) and subsequently refined with Muscle 3.8.425 to minimize gaps and mismatch interference. Given that *AGL6* is a hallmark of the MIKC^c^-type MADS-box family, characterized by the dual presence of “MIKC^c^-type” MADS-box and K-box domains, we employed the SMART database [43] for rigorous domain architecture prediction. Only sequences concurrently harboring the MADS-box domain (InterPro ID: IPR033896) and the K-box domain (PFAM ID: PF00319) were designated as core members of the *AGL6* family for downstream analysis.

**Figure 1:**
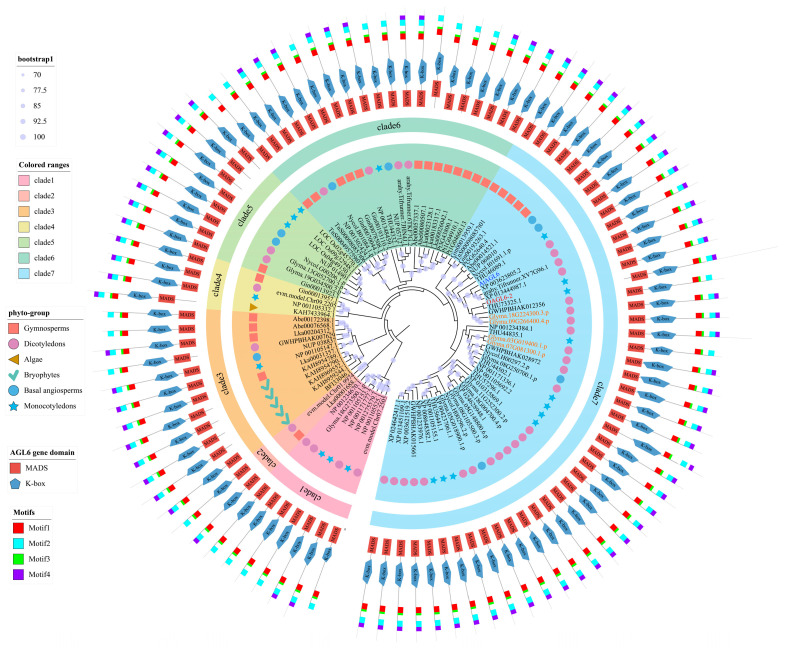
Circular phylogenetic tree and multidimensional annotation of the *AGL6* gene family across representative plant groups. The phylogenetic tree was constructed using the Maximum Likelihood (ML) method via IQ-TREE 2.0, with JTT + R7 identified as the optimal protein substitution model and 1000 bootstrap replicates. Colored bars at the branch tips indicate bootstrap support values (Legend: bootstrap1; The circles on the evolutionary branches go from small to large corresponds to the 70–100 support range). The concentric layers (from inner to outer) display the following information: *AGL6* homolog identifiers (e.g., *OAGL6-2*); colored blocks representing seven distinct evolutionary clades (Legend: Colored ranges); symbols and colors denoting different plant taxonomic groups (Legend: phyto-group, e.g., triangles for *algae* and circles for *gymnosperms*); red squares and blue pentagons indicating the conserved MADS-box and K-box domains of the *AGL6* genes, respectively (Legend: *AGL6* gene domain, where MADS represents the MADS-box domain and K-box represents the K-box domain); and multicolored bars illustrating the distribution of four conserved protein motifs (Legend: Motifs, i.e., Motif 1–4).

Based on the identified 98 *AGL6* homologs, a Maximum Likelihood (ML) phylogenetic tree was constructed using IQ-TREE 2.0. The ModelFinder module identified JTT + R7 as the optimal substitution model, and branch reliability was assessed via a 1000-replicate bootstrap test. The resulting phylogeny (Figure 1) clearly partitioned all *AGL6* candidates into seven distinct evolutionary clades (Clades 1–7, indicated by colored regions). Most branches exhibited bootstrap support values ≥ 70%, confirming the robustness of the tree topology.

To further validate the classification of these clades, motif prediction was performed using MEME 5.5.3 (maximum motifs = 4). All identified *AGL6* homologs were found to contain Motif 1 to Motif 4, which, upon annotation, were verified to correspond to the core functional regions of the MADS-box and K-box domains, respectively. These results are highly congruent with the domain predictions from SMART and PFAM, supporting the structural conservation of the family across lineages.

### 2.2. Identification and Phylogenetic Classification of Candidate GmAGL6 Genes

**Figure 2:**
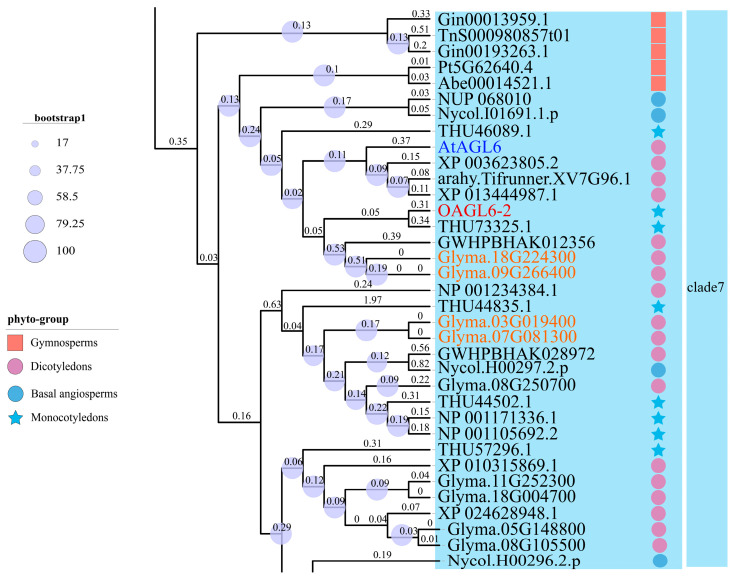
Magnified phylogenetic sub-tree of the core AGL6 lineage for the identification of soybean candidates. This figure represents a high-resolution, magnified view of Clade 7, derived from the global AGL6 phylogenetic tree shown in Figure 1. The sub-tree highlights the phylogenetic positioning of the four soybean candidate genes (orange labels) relative to the orchid homolog OAGL6-2 (red label) and *Arabidopsis* AtAGL6 (blue label). The diameter of the circles at the internal nodes represents bootstrap support values based on 1000 replicates. Numerical values on the branches indicate branch lengths, reflecting the degree of sequence divergence. Identical protein sources and taxonomic symbols are used here as in Figure 1 to maintain consistency.

Based on the global multi-species phylogenetic tree constructed in Section 2.1 (Figure 1), we performed a targeted screening for soybean *AGL6* homologs. To facilitate a more precise observation of the evolutionary distances and branch supports among the core members, the lineage containing *OAGL6-2* and its closest relatives (designated as Clade 7 in Figure 1) was extracted and magnified to generate a detailed sub-tree (Figure 2). This focused analysis revealed that four genes in the soybean genome (*Glyma.18G224300*, *Glyma.09G266400*, *Glyma.03G019400* and *Glyma.07G081300*) clustered into a high-confidence monophyletic group with *OAGL6-2* (bootstrap support ≥ 89%). These results indicate that the aforementioned soybean genes share a close phylogenetic relationship and a highly conserved evolutionary origin with *OAGL6-2*, suggesting that these four soybean genes are bona fide members of the *AGL6* subfamily and may participate in soybean floral development. Consequently, these four genes were tentatively designated as *GmAGL6a* (*Glyma.09G266400*), *GmAGL6b* (*Glyma.18G224300*), *GmAGL6c* (*Glyma.03G019400*), and *GmAGL6d* (*Glyma.07G081300*) as candidate members of the soybean *AGL6* gene family (Table 1).

**Figure 3:**
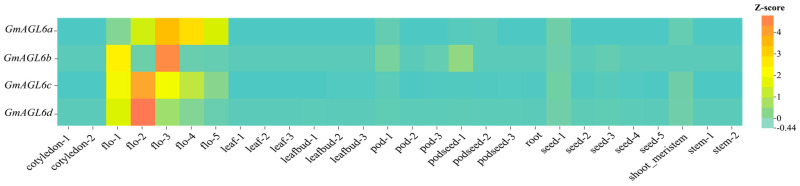
Tissue-specific expression heatmap of *GmAGL6a–d* in the W82.a4.v1 background. This heatmap displays the relative expression profiles of four soybean *AGL6* candidate genes (*GmAGL6a–d*) across distinct tissues and fine-scale developmental sub-periods. Expression data were obtained from the SoyOD database and normalized using Z-scores. The color bar on the right indicates Z-score values (red = high relative expression; teal = low relative expression). The abscissa labels correspond to specific soybean growth and developmental sub-periods as follows: cotyledon-1, early germination stage (cotyledon expansion period); cotyledon-2, late germination stage (functional cotyledon period); flo-1 to flo-5, five sequential sub-periods of flower bud development (from bud initiation to bud maturation); leaf-1, leaf-2, leaf-3, leaf organ developmental sub-periods during flowering; leafbud-1, leafbud-2, leafbud-3, leaf bud differentiation sub-periods during flowering; pod-1, pod-2, pod-3, three sequential sub-periods of young pod formation; podseed-1, podseed-2, podseed-3, three transition sub-periods from pod development to seed initiation; seed-1 to seed-5, five sequential sub-periods of seed development (from immature seed to mature seed); root, root tissue during vegetative growth; shoot_meristem, shoot apical meristem; stem-1, stem-2, two sequential elongation sub-periods of stem development. Notably, *GmAGL6a–d* exhibit relatively high expression in flowering stage-related tissues, which aligns with the functional conservation of *AGL6* subfamily genes in regulating floral development.

To further corroborate the functional relevance of the identified candidates, FPKM expression values for the four genes across diverse tissues and organs of the Williams 82 cultivar (W82.a4.v1 genome) were retrieved from the Soybean Omics Database (SoyOD) [44]. Following Z-score normalization of the raw data from three biological replicates, a tissue-specific expression heatmap was generated (Figure 3). The expression profiles indicated that all four *GmAGL6* genes are either minimally expressed or entirely silenced in vegetative organs, including roots (R1), stems (S1), and leaves (L1). In contrast, they exhibit significant transcript enrichment during key stages of floral organogenesis, representing a distinct floral-specific expression pattern. This characteristic aligns with the functionally conserved “floral-preferential expression” of *AGL6* orthologs reported in *Arabidopsis*, tomato, and rice, which is consistent with previously reported floral-preferential expression patterns of *AGL6* orthologs in other angiosperms and supports their potential involvement in soybean floral development.

Furthermore, domain architecture analysis (Section 2.1) confirmed that the four candidate genes harbor intact MADS-box (InterPro ID: IPR033896) and K-box (PFAM ID: PF00319) conserved domains, which serve as diagnostic structural signatures for the MIKC^c^-type *AGL6* subfamily. To further resolve the sequence characteristics and evolutionary conservation of GmAGL6 proteins at the amino acid level, a comprehensive multiple sequence alignment was performed comparing the four soybean GmAGL6 proteins (*GmAGL6a–d*) with the orchid *OAGL6-2*. The alignment results (Figure S1) revealed high amino acid sequence conservation among the four soybean AGL6 candidates, particularly within the regions defining the typical structural framework of MIKC^c^-type MADS-box proteins.

This structural integrity, coupled with phylogenetic homology and tissue-specific expression trends, provides robust evidence for the classification of *GmAGL6a–d* as the core constituents of the soybean *AGL6* gene family. Their evolutionary conservation and marked floral-specific expression strongly implicate these genes in the regulatory network of soybean floral development. Consequently, these four genes were prioritized for subsequent CRISPR/Cas9-mediated gene editing and functional validation experiments.

### 2.3. Generation and Characterization of CRISPR/Cas9-Mediated Soybean AGL6 Quadruple Mutants

To investigate the specific roles of the four candidate *GmAGL6* homologs in regulating soybean floral architecture and keel petal fusion, a CRISPR/Cas9-mediated gene editing strategy was employed. We designed two sgRNA target sites for each member, totaling eight targets across the four genes. Notably, the targets for *GmAGL6a* and *GmAGL6b* were localized within the MADS-box domain, whereas the targets for *GmAGL6c* and *GmAGL6d* were situated in the MADS-box and K-box domains, respectively (Table 2). These target sequences were cloned into a linearized CRISPR/Cas9 vector to construct a recombinant multi-target editing system. Soybean genetic transformation was performed using *Agrobacterium* tumefaciens strain *EHA105*, following the methodology described in [45].

Transformation yielded three independent T0 generation quadruple mutant lines (designated as *Gmagl6-1*, *Gmagl6-2*, and *Gmagl6-3*). Sanger sequencing of the flanking genomic regions confirmed that all editing events occurred precisely upstream of the PAM sequences, validating the efficacy of the CRISPR/Cas9 system. Genotypic analysis revealed that in the *Gmagl6-1* line, deletions were introduced at the first target site for all four *GmAGL6* genes. In contrast, mutations were not detected at the first target sites for *GmAGL6c* and *GmAGL6d* in the *Gmagl6-2* and *Gmagl6-3* lines (Figure S2).

Protein structure modeling and prediction using SWISS-MODEL indicated that the mutations in the *Gmagl6-1* line resulted in protein truncations, leading to the loss of functional MADS-box or K-box domains. In the *Gmagl6-2* and *Gmagl6-3* lines, mutations led to the truncation of the MADS-box domains in *GmAGL6a* and *GmAGL6b*, as well as the K-box domains in *GmAGL6c* and *GmAGL6d* (Figure S3). These structural variations were predicted to cause abnormal protein conformations in the respective domains for all candidate proteins.

The three T0 quadruple mutant lines were allowed to self-pollinate to obtain T1 progeny. However, seeds harvested from the *Gmagl6-1* line failed to germinate, precluding the acquisition of T1 plants. For the *Gmagl6-2* and *Gmagl6-3* lines, the editing status of the target sites in T1 plants was verified via sequencing. The results showed that the Sanger sequencing chromatograms for *GmAGL6c* and *GmAGL6d* in T1 plants of the *Gmagl6-2* line were clean single peaks identical to the wild-type (Williams 82) sequence. This indicates that the CRISPR/Cas9 system failed to introduce stable mutations at these specific loci in the *Gmagl6-2* line, which retained a wild-type (Williams 82) genotype. Consequently, the *Gmagl6-2* line was not progressed to the T2 generation or used for phenotypic analysis.

Significant variations in seed germination capacity were observed among the three independent T0 mutant lines obtained in this study. Specifically, seeds from the *Gmagl6-1* line exhibited a total lack of germination after harvest, suggesting that embryo development or seed viability may have been severely compromised. Sequence analysis identified *Gmagl6-1* as the sole line harboring effective edits in the MADS-box domains (Target 1) of both *GmAGL6c* and *GmAGL6d*. In contrast, *Gmagl6-2* retained the wild-type (Williams 82) genotype for *GmAGL6c/d*, while *Gmagl6-3*, despite being mutated at these loci, carried edits localized within the K-box domain (Target 2) rather than the MADS-box region. This correlation between genotype and phenotype provides a preliminary indication that the structural integrity of the *GmAGL6c* and *GmAGL6d* MADS-box domains may be essential for proper seed germination in soybean.

Therefore, all subsequent phenotypic characterizations of the *AGL6* mutants were conducted using the T2 population derived from the *Gmagl6-3* line.

### 2.4. Impact of Gmagl6 Quadruple Mutants on Soybean Floral Organ Morphology

To systematically evaluate the regulatory role of the *GmAGL6* gene family on soybean floral phenotypes, the T2 homozygous population of the *Gmagl6-3* mutant line was selected as the research material. All experimental materials were genotyped via Sanger sequencing of the target sites to ensure stable homozygous inheritance across the four homologous loci.

**Figure 4:**
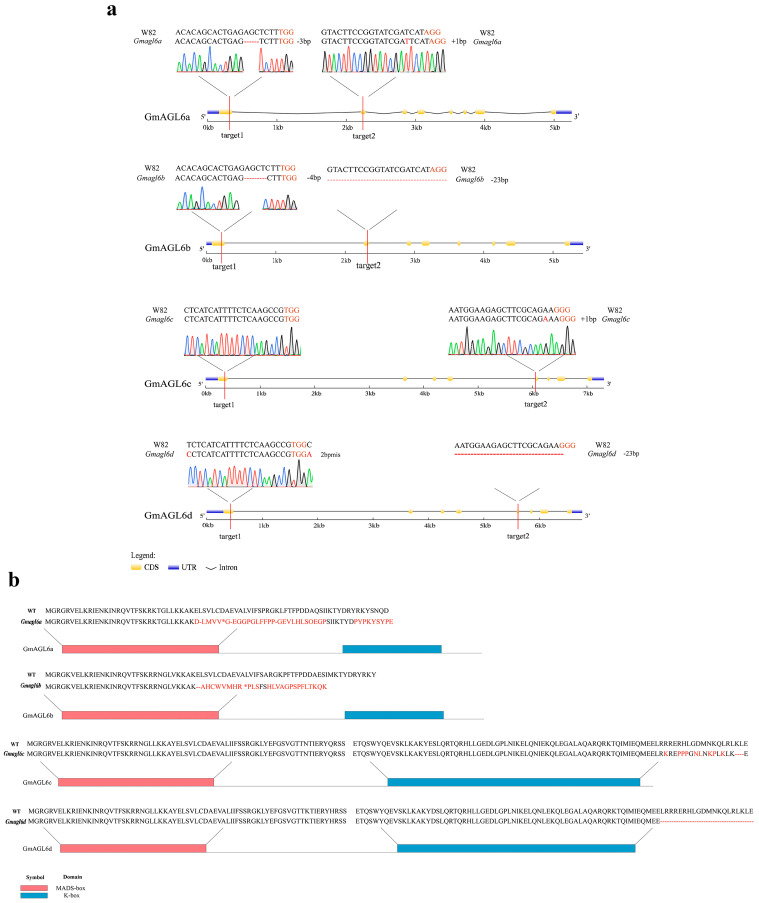
Gene editing profiles of *Gmagl6* T2 generation mutants and deduced amino acid sequence alignment of *Gmagl6* between wild-type (Williams 82) and gene-edited lines. (**a**) Genomic sequence alignment of the target region between the wild type (WT) and the T2 mutant. The PAM sequence is shown in dark red, and mutated nucleotides are highlighted in bright red. The hyphen (“-”) indicates nucleotide deletions. The Sanger sequencing chromatogram is shown below to confirm the mutation. (**b**) Alignment of the predicted amino acid sequences of WT and the mutant. Altered amino acids are highlighted in bright red. The hyphen (“-”) indicates amino acid deletions, and “*” indicates a premature stop codon.

Genotypic analysis revealed specific allelic variations within this mutant line: in *GmAGL6a*, the two target sites exhibited a 3-bp deletion and a 1-bp insertion, respectively; *GmAGL6b* harbored a 4-bp deletion at the target site; in *GmAGL6c*, the first target site remained unedited, while the second site introduced a 1-bp insertion; *GmAGL6d* displayed a 2-bp mismatch at the first target site located 1-bp downstream of the PAM sequence, with a base deletion at its second target site. Aside from the first target of *GmAGL6d*, all detected editing events were localized within the typical cleavage regions upstream of the PAM. The Sanger sequencing chromatograms for all target sites presented clean, symmetrical single-peak signals, indicating high genotypic consistency within the mutant population and excluding the possibility of interference from chimerism or multi-allelic editing (Figure 4).

Furthermore, to exclude the interference of potential off-target effects on the experimental conclusions, we strictly validated the highest-scoring potential off-target site for the shared target 1 of *GmAGL6c* and *GmAGL6d* (located in the coding region of the *GLYMA16G03097* gene, with an off-score of 0.61). Utilizing the Sanger Sequencing Result Check tool in TBtools v2.458 [46], we performed dual-strand sequence alignment and chromatogram analysis on the mutant population. The results confirmed that the *GLYMA16G03097* gene remained in a homozygous wild-type (Williams 82) state across all samples used for floral and phenotypic analysis, with no non-targeted editing events detected (Figure S4). This evidence supports the conclusion that the observed phenotypes in the *Gmagl6* quadruple mutants were primarily caused by targeted editing of the intended genes rather than by the tested off-target effect.

**Figure 5:**
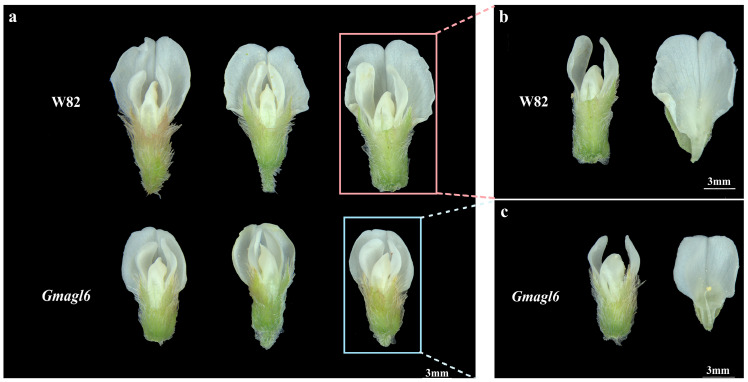
Morphological and structural characterization of floral organs in *Gmagl6* mutants and wild-type (Williams 82). (**a**) Overall floral morphology of wild-type (Williams 82) (**upper row**) and *Gmagl6* mutants (**lower row**). Pink and blue boxes indicate the individual flowers magnified in panels (**b**) and (**c**), respectively. (**b**) Magnified view of a Williams 82 flower with the standard petal removed, showcasing the organized arrangement of internal floral organs. (**c**) Magnified view of a *Gmagl6* quadruple mutant flower with the standard petal removed. Photographs were captured at full anthesis from representative homozygous individuals. These images are presented to highlight the retention of keel petal identity and fusion in the mutant flowers. Although representative flowers showed slight variation in outline, quantitative analysis did not detect significant differences in flower height or width, whereas the flower height-to-width ratio was significantly increased in *Gmagl6* mutants.

Regarding floral morphology, the *Gmagl6* quadruple mutants maintained overall structural consistency with the wild-type (Williams 82), exhibiting the typical papilionaceous floral structure of soybean, which includes one standard petal, two wing petals, and two fused keel petals. No organ loss or changes in organ number were observed in the mutant flowers, and the spatial arrangement of the floral organs remained unaltered (Figure 5a).

Further observation indicated that the standard, wing, and keel petals of the mutants were morphologically similar to those of the wild-type (Williams 82) in their relative positions. The keel petals successfully enclosed the stamens and pistil, with their fusion zones and bilateral symmetry remaining intact (Figure 5b,c). These findings suggest that mutations in the *AGL6* gene family do not disrupt the fundamental architecture of the floral organs.

**Figure 6:**
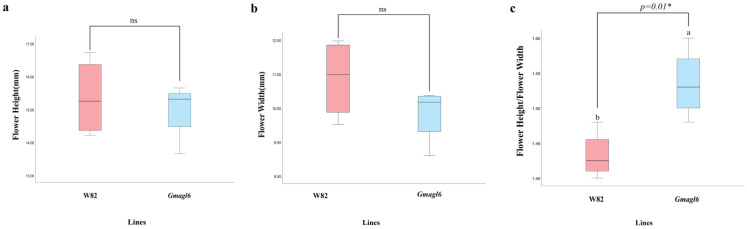
Statistical comparison of floral morphological traits between wild-type (Williams 82) and *Gmagl6* quadruple mutants. (**a**) Flower height; (**b**) Flower width; (**c**) Flower height/flower width ratio. Data are presented as boxplots showing the median, quartiles, and range (Williams 82, *n* = 4; *Gmagl6*, *n* = 4), where *n* indicates the number of independent biological replicates. Different letters (e.g., a and b) indicate significant differences between groups. Statistical significance was determined by an independent-samples *t*-test. ns, not significant; “*” indicates a significant difference at *p* = 0.01.

To further quantify floral morphology, we measured flower height, flower width, and the flower height-to-width ratio in wild-type (Williams 82) and *Gmagl6* quadruple mutants (Figure 6, Tables S5–S7). Statistical analysis showed that flower height and flower width did not differ significantly between the two genotypes. However, the flower height-to-width ratio was significantly higher in the *Gmagl6* mutants than in the wild type (*p* = 0.01), indicating that *GmAGL6* mutation primarily affected floral proportion rather than overall floral size.

### 2.5. Effects of GmAGL6 Quadruple Mutants on Vegetative Growth, Podding, and Seed Traits

Following the floral phenotypic characterization, we further evaluated the vegetative and reproductive performance of the T2 population derived from the self-pollinated *Gmagl6-3* quadruple mutant line in comparison with the wild-type (Williams 82).

**Figure 7:**
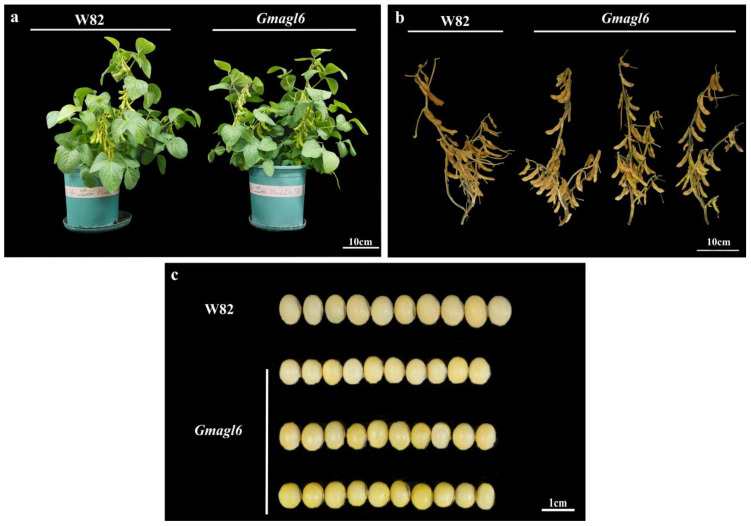
Phenotypic comparison of plant architecture, mature pod morphology, and seed traits between the wild-type (Williams 82) and *Gmagl6* mutant lines. (**a**) Morphological characteristics of mature Williams 82 (W82, **left**) and *Gmagl6* (**right**) plants, highlighting the overall vegetative growth performance of the two materials. (**b**) Mature pod morphology of the Williams 82 (W82, **left**) and *Gmagl6* (**right**) lines. (**c**) Seed phenotypes of the Williams 82 (W82, **upper row**) and *Gmagl6* (**lower two rows**) lines.

During the vegetative growth phase, no discernible differences were observed in the overall plant architecture between the *Gmagl6* quadruple mutants and the wild-type (Williams 82). Key traits, including plant height, branching patterns, and leaf morphology and coloration, remained consistent across both genotypes. The mutant plants exhibited normal growth without evidence of developmental stunting or significant aberrations (Figure 7a). In the late stages of reproductive development, the *Gmagl6* quadruple mutants successfully completed flowering and pollination, ultimately forming normal pods. The pod morphology, length, and spatial arrangement in the mutants mirrored those of the wild-type (Williams 82), with no significant defects in podding capacity or pod development detected (Figure 7b). Comparative analysis of mature seeds revealed that the seeds of the *Gmagl6* quadruple mutants were morphologically typical, with intact seed coats and no evident abortion or deformity. However, a significant reduction in overall seed size was observed in the mutants compared to wild-type (Williams 82) (Figure 7c). This small-seed phenotype was reproducible across multiple T2 mutant individuals, manifesting as a collective reduction in seed dimensions rather than sporadic abnormalities in isolated individuals.

Collectively, these results demonstrate that while the *AGL6* quadruple mutation does not substantially alter the overall vegetative growth or podding efficiency of soybean, it exerts a specific influence on seed development and final size.

**Figure 8:**
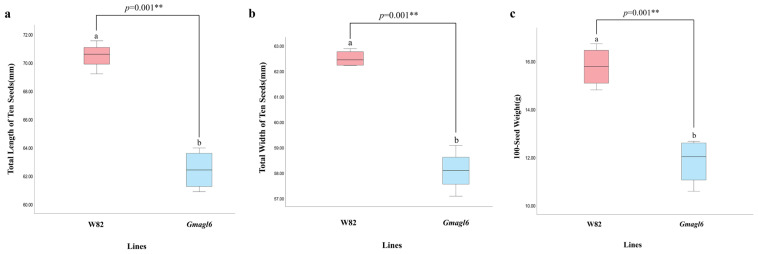
Statistical comparison of seed morphological traits between wild-type (Williams 82) and *Gmagl6* quadruple mutants. (**a**) Total length of ten seeds; (**b**) Total width of ten seeds; (**c**) 100-seed weight. Data are presented as boxplots highlighting the median, quartiles, and range (*n* = 5). Different letters (e.g., a and b) indicate significant differences between groups. Statistical significance was determined by an independent-samples *t*-test. **, *p* < 0.01.

Quantitative assessment further corroborated the small-seed phenotype observed in the *Gmagl6* quadruple mutants (Figure 8, Tables S8–S10). Statistical comparisons revealed that both the total length and total width of ten seeds in the mutant lines were significantly reduced compared to those of the wild-type (Williams 82). Furthermore, a marked decrease in the 100-seed weight was observed in the *Gmagl6* mutants. These results demonstrate that the loss of *GmAGL6* function leads to a consistent and systemic reduction in seed scale and mass across multiple dimensions.

## 3. Discussion

### 3.1. Evolutionary and Structural Perspectives on GmAGL6 Genes in Soybean

The identification of four *GmAGL6* homologs (*GmAGL6a–d*) in soybean, each characterized by the co-occurrence of the MADS-box and K-box domains, provides a modern genomic perspective on a lineage with deep evolutionary roots. Members of the AGL6 subfamily belong to the MIKC^c^-type MADS-box transcription factors, which were historically considered to be specific to land plants. However, recent phylogenomic studies suggest that the structural framework of MIKC^c^-type MADS-box proteins—and the emergence of the K-domain itself—predates the origin of land plants, extending back to early streptophyte lineages including *charophycean* green algae [47]. This evolutionary scenario implies that the ancestral molecular components underlying *AGL6*-related regulatory mechanisms were already present prior to the terrestrialization of plants.

To place soybean *AGL6* genes within a broader evolutionary context, representative species from early-diverging plant lineages were included in the phylogenetic analysis, including the green alga *Chlorella* and the bryophyte *Marchantia polymorpha*. Although the sequences identified from these taxa do not belong to the *AGL6* clade, homologous genes retrieved through HMMER searches were retained in the phylogenetic tree. Domain and motif analyses further revealed that these proteins contain conserved MADS-box and K-box domains, as predicted by SMART v10 (Simple Modular Architecture Research Tool; https://smart.embl.de/) and MEME analyses, consistent with previous findings that key structural components of MIKC^c^-type MADS-box transcription factors were already present in early streptophyte lineages [47]. These observations suggest that while the *AGL6* subfamily itself likely emerged later during the evolution of seed plants, the underlying MIKC^c^-type structural framework had already undergone significant evolutionary establishment in earlier plant ancestors.

The structural conservation of *GmAGL6a–d* is particularly noteworthy when viewed through the lens of protein architecture evolution. Previous studies indicate that ancestral MIKC^c^-type MADS-box proteins (such as those identified in charophytes) often possess relatively long C-terminal regions, resulting in compact “sphere-like” conformations that may have limited their interaction capacity to simple dimers. A key evolutionary innovation in land plant MIKC^c^-type MADS-box proteins was the shortening of the C-terminus, which exposed the K-domain and enabled the formation of higher-order tetrameric complexes—the structural basis of the well-established “floral quartet” model [11,47]. The conserved domain architecture observed in soybean *GmAGL6* proteins therefore suggests that they participate in the complex transcriptional regulatory networks characteristic of angiosperm floral development.

Consistent with this evolutionary framework, transcriptional profiling revealed strong enrichment of *GmAGL6* expression during flower bud differentiation and floral organ maturation, mirroring the floral-preferential expression patterns reported for AGL6 homologs in diverse angiosperms such as rice and orchids [24,40]. Interestingly, although the *Gmagl6* quadruple mutants in soybean retain the fundamental papilionaceous floral architecture without the homeotic transformations commonly observed in monocots, they exhibit altered floral proportion and reduced seed size. This phenotypic divergence suggests that the *AGL6* lineage in legumes may have undergone functional specialization, shifting from a primary role in floral organ identity determination toward the regulation of reproductive organ proportion and seed development. Such lineage-specific functional diversification likely reflects the broader evolutionary trajectory of MIKC^c^-type MADS-box genes, in which ancient structural frameworks have been repeatedly co-opted and refined to regulate the complex floral morphologies observed across flowering plants.

### 3.2. Functional Redundancy and Divergence of GmAGL6 in Floral Organ Identity

Despite the high homology between *GmAGL6* and orchid *OAGL6-2*, our CRISPR/Cas9-mediated quadruple mutants (*Gmagl6*) did not exhibit the anticipated loss of the keel or homeotic transformations. In addition, the likelihood that the phenotypes observed in *Gmagl6* were caused by detectable off-target mutations appears to be low. Among the predicted candidate off-target loci, *GLYMA16G03097* had the highest off-target score (0.61). Nevertheless, no mutation was detected at this site by targeted amplification and sequencing. Although the possibility of undetected extremely low-frequency off-target events cannot be completely excluded, these data support the view that the major phenotypes described here are mainly associated with disruption of the *GmAGL6* genes. This result stands in stark contrast to monocot studies, where *Osmads6* mutants in rice lose palea and lodicule identity, and silencing *OAGL6-2* in orchids leads to the complete reversion of the labellum into sepal/petal-like structures.

Two primary factors may account for this phenotypic divergence. First is functional redundancy. As a paleopolyploid crop, the soybean genome contains a vast number of duplicated genes. Within the MADS-box framework, members of the *SEPALLATA (SEP)* subfamily (E-class genes) frequently form heteromultimeric complexes with *AGL6* to coordinate floral development. In *Petunia*, single *phagl6* mutants show no discernible defects in floral or ovule morphology; a complete sepaloid transformation of petals only occurs when both *PhAGL6* and *FBP2* (an E-class gene) are simultaneously mutated [12,15]. Thus, it is highly probable that other MADS-box genes in soybean, such as *GmSEP-like* members, compensate for the loss of *GmAGL6* to maintain fundamental floral identity. Second is functional divergence. Despite sequence conservation, the core role of *AGL6* in dicots may have transitioned from “organ identity determination” to “fine-tuning” developmental processes. In *Arabidopsis*, *AGL6* primarily acts as a regulator of flowering time and lateral organ development rather than a primary identity determinant [14]. Notably, the *Cymbidium* genome analysis further demonstrated the potent role of *AGL6*-like genes in organ identity determination; their ectopic expression in leaves can even trigger perianth-like morphological transformations [34]. Our results suggest that in the specialized papilionaceous flowers of soybean, suggesting that keel identity in soybean may depend more strongly on other floral regulatory pathways than on the *AGL6* lineage, reflecting divergent evolution in floral morphogenesis between legumes and orchids.

### 3.3. GmAGL6 Affects Floral Proportion and Seed Size

The most prominent phenotype observed in the *Gmagl6* quadruple mutants was a significant reduction in mature seed size, together with a significant increase in the floral height-to-width ratio. In contrast, flower height and width were not significantly different between the mutant and wild-type plants. These results suggest that *GmAGL6* may influence floral proportion rather than absolute floral size, while playing a more evident role in seed development. We hypothesize that *GmAGL6* genes may contribute to the regulation of reproductive organ proportion and seed development rather than directly specifying their organ type. This is consistent with earlier findings in *Arabidopsis* and *Chimonanthus praecox*, where *AGL6-like* genes were shown to influence cell division, organ expansion, and flowering time [14,18]. In our study, the number and arrangement of floral organs remained normal in the quadruple mutants, whereas the floral height-to-width ratio was significantly increased. This suggests that *GmAGL6* may modulate floral proportion rather than overall floral size. Furthermore, unlike the maize and wheat *AGL6* homologs that affect meristem determinacy, *GmAGL6* in soybean appears to influence floral proportion and seed development without disrupting floral organ identity. Furthermore, while mutations in the maize *AGL6* homolog *ZAG3* and wheat *AGL6* impact meristem determinacy [25,26], our results in soybean showed an alteration in floral proportion rather than an overall reduction in floral size rather than meristematic proliferation. These results suggest that *GmAGL6* may be involved in the regulation of floral proportion and seed development in soybean. The reduction in seed size observed in the mutants further supports a role for *GmAGL6* in reproductive development, although the underlying molecular mechanisms remain to be clarified. This phenotypic pattern is consistent with the broader role of MADS-box transcription factors as regulators within complex developmental networks. It also agrees with previous reports that *AGL6* homologs are expressed in grass ovules and participate in their development [23], indicating that the *AGL6* lineage may function in multiple reproductive developmental processes beyond classical floral homeotic regulation.

Besides, in our research, the lethal phenotype of the *Gmagl6-1* line among the T0 generation of soybean *GmAGL6* gene-edited lines provides a novel perspective for understanding the functional thresholds of this gene family. Structurally, the MADS-box domain is primarily responsible for sequence-specific DNA binding, while the K-box mediates essential protein–protein interactions. The inactivation of the MADS-box domains in *GmAGL6c* and *GmAGL6d* within the *Gmagl6-1* line likely led to a complete disruption of transcriptional regulation for downstream targets vital for seed development; in contrast, the mutations in *Gmagl6-3* involved only partial truncations of the K-box, which may have preserved sufficient basal transcriptional activity to support germination, albeit resulting in a reduced seed size phenotype. Since the *Gmagl6-1* seeds failed to germinate, we were unable to perform repeated validation in subsequent generations. Consequently, it remains undetermined whether this phenomenon stems from the specific loss of *GmAGL6c/d* function or represents a “dosage accumulation effect” arising from the quadruple mutations. These preliminary observations highlight a potential role for *GmAGL6* in seed germination, and it is necessary to develop more refined allelic series in the future to clarify the precise contribution of the MADS-box domain to this process.

### 3.4. GmAGL6 Functions as a Quantitative Regulator of Floral Proportion and Seed Size

Our quantitative data indicate that *GmAGL6* primarily affects seed size and floral proportion in soybean. Although flower height and width were not significantly altered, the flower height-to-width ratio was significantly increased in the mutants. Together with the reduction in seed-related traits, these findings suggest that *GmAGL6* functions in fine-tuning floral proportion and seed development rather than directly controlling floral organ identity or absolute floral size. This role differs from that reported in monocots. For example, in rice and orchids, loss of *AGL6* orthologs typically causes homeotic transformations or the loss of specialized organs such as the palea or labellum. In contrast, in the papilionaceous flowers of soybean, the four petals (standard, wings, and keels) remained correctly positioned and morphologically typical in the quadruple mutants, despite the significant change in floral proportion. This divergence suggests that, during legume evolution, the *AGL6* lineage may have been co-opted for the quantitative regulation of floral proportion and seed development, diverging from its ancestral role in organ specification. The altered floral proportion observed in the mutants further suggests that *GmAGL6* may influence growth-related processes, potentially involving cell expansion or the regulation of developmental boundaries in reproductive tissues. Given that seed weight is a critical component of soybean yield, the significant reduction in 100-seed weight observed in our mutants further suggests that *GmAGL6* may represent a potential target for genetic improvement of seed size and yield-related traits.

### 3.5. Limitations and Perspectives

This study demonstrates, via CRISPR/Cas9-mediated gene editing, the significant role of the *GmAGL6* gene family in regulating the size of soybean flowers and seeds. However, its potential contribution to floral organ identity may be masked by functional redundancy. The construction of higher-order mutants combining *AGL6* with other MADS-box genes (e.g., *GmSEP*) will be essential to further unravel the genetic networks governing soybean floral identity. Additionally, identifying protein interactors (such as *GmSEP* or *GmAP1* homologs) and utilizing RNA-seq to identify differentially expressed genes in flower buds and young seeds between mutant and wild-type (Williams 82) plants, will help elucidate the downstream regulatory networks through which *GmAGL6* coordinates cell growth and seed development.

## 4. Materials and Methods

### 4.1. Identification and Phylogenetic Analysis of AGL6 Homologs in Glycine max

To identify *AGL6* homologs in the soybean genome (Wm82.a4.v1), the amino acid sequence of the orchid AGL6 protein, OAGL6-2 (GenBank: ADJ67237.1), was utilized as a query for homology searches using HMMER v3.3.2, with an E-value threshold set to 1 × 10^−4^. Soybean genomic sequences and corresponding annotation data were retrieved from the Phytozome v13 database (https://phytozome-next.jgi.doe.gov/, accessed on 10 December 2025). The domain architecture of the resulting candidate proteins was predicted using the SMART analysis tool [43]; only sequences simultaneously harboring both MADS-box and K-box domains were retained for subsequent analyses.

To determine the phylogenetic position of these soybean candidates, *AGL6* orthologous sequences were collected from diverse representative plant species. These included monocots (e.g., *Oryza sativa*, *Zea mays*, *Musa acuminata*), dicots (e.g., *Arabidopsis thaliana*, *Solanum lycopersicum*), legumes (e.g., *Arachis hypogaea*, *Cercis canadensis*, *Medicago truncatula*), basal angiosperms (*Nymphaea caerulea*, *Nymphaea thermarum*, *Amborella trichopoda*), and gymnosperms (e.g., *Gnetum montanum*, *Ginkgo biloba*, *Larix kaempferi*, *Pinus tabuliformis*, *Abies alba*), algae (*Chlorella vulgaris*), bryophytes (e.g., *Marchantia polymorpha*). Protein sequences were obtained from NCBI, Phytozome v13, and UniProt (https://www.uniprot.org/, accessed on 10 December 2025).

Multiple sequence alignments were performed using MAFFT v7.475 [48] and Muscle [49]. Phylogenetic trees were constructed using the Maximum Likelihood (ML) method in IQ-TREE v2.2.0 [7], with the optimal substitution model automatically selected based on the Bayesian Information Criterion (BIC). Branch support was evaluated using 1000 bootstrap replicates. The generated phylogenetic trees were subsequently uploaded to the iTOL (Interactive Tree of Life) web server [50] for visualization and branch annotation.

Conserved motifs within AGL6 proteins were predicted using the MEME Suite (v5.5.9) [51], with the maximum number of motifs set to 4 and all other parameters at default settings. Predicted motifs were annotated in conjunction with known MADS-box and K-box domain information, the boundaries of which were further confirmed using the SMART and PFAM databases. Final alignments were visualized using ESPript 3.0 [52].

The phylogenetic classification of soybean *AGL6-like* genes was determined based on the constructed tree. Subsequently, tissue-specific expression data were extracted from the soybean developmental transcriptome database, SoyOD [44]. FPKM values across various tissues and developmental sub-stages of the W82.a4 genome were retrieved and subjected to Z-score normalization based on three biological replicates. The normalized data were utilized to generate heatmaps to characterize the expression patterns of *GmAGL6* genes during soybean growth and development.

### 4.2. Plant Materials and Growth Conditions

Soybean cultivar Williams 82 (W82) was used as the wild-type (WT) control. All soybean seeds Germination was conducted on moist filter paper under controlled greenhouse conditions, featuring a constant temperature of 24 °C and a 16/8 h light/dark cycle. Seedlings were maintained until the cotyledons became green and the hypocotyl length reached approximately 4 cm. By this stage, a well-developed primary root system had formed, accompanied by the initiation of lateral roots. Seedlings were then transplanted into 50-hole nursery trays containing a mixture of nutrient soil and vermiculite (3:1, *v*/*v*). Plants were initially grown in a greenhouse under standard conditions (28 °C day/24 °C night, 16 h light/8 h dark). Upon the emergence of two pairs of true leaves, the plants were moved to larger pots and maintained under outdoor conditions in Sanya, Hainan Province from September to November.

### 4.3. CRISPR/Cas9 Vector Construction and Plant Transformation

To achieve simultaneous mutagenesis of the four soybean *AGL6* homologs (*GmAGL6a–d*), four sgRNA targets were designed using the CRISPR-P 2.0 online tool. Given the high sequence conservation among the homologs, a “multi-targeting” strategy was adopted: two sgRNAs were designed to target conserved sequences in both *GmAGL6a* and *GmAGL6b* within the functionally critical MADS-box domain; the remaining two sgRNAs targeted shared regions in the first or second exons of *GmAGL6c* and *GmAGL6d*, corresponding to the MADS-box and K-box domains, respectively. Target selection strictly adhered to established criteria: priority was given to sequences located in the proximal coding region with low predicted off-target scores and high editing efficiency, while excluding regions containing introns or splice sites (Supplementary Tables S1–S4). The four selected sgRNAs were integrated into a modified CRISPR/Cas9 expression vector, pCAMBIA3300 (carrying the *Bar* selection marker), via Golden Gate cloning to assemble a multi-target co-editing system.

The recombinant plasmids were introduced into Williams 82 via *Agrobacterium*-mediated cotyledonary node transformation, following the protocol described by Song et al. [53]. This process involved cotyledonary node pre-culture, *Agrobacterium* infection, selective culture, and plant regeneration. Positive T0 transformants were identified using an immunochromatographic assay to detect the expression of the Bar protein. Briefly, leaf tissue was sampled from the transplanted seedlings and processed using the PAT/bar Colloidal Gold Test Strip Kit #RIA03 (BioRun Biotechnology Co., Ltd., Wuhan, Hubei, China) according to the manufacturer’s instructions. The resulting protein extract was incubated with the test strip for 5–10 min at room temperature. Seedlings exhibiting distinct chromogenic bands at both the control (C) and test (T) lines were identified as Bar-positive individuals harboring the CRISPR/Cas9 vector. These results were subsequently confirmed through PCR amplification of the *Bar* gene. Positive transformants were confirmed further via PCR amplification of the *Bar* gene.

Genotypic analysis of *Bar*-positive T0 seedlings was conducted using PCR with primers flanking the target sites, followed by Sanger sequencing. Sequence alignments were performed to identify mutation types (e.g., insertions, deletions, frameshifts, or premature terminations) and confirm functional knockouts.

### 4.4. Phenotypic Characterization of Floral Organs

Floral organs were collected from homozygous T2 generation *Gmagl6* quadruple mutants and wild-type (Williams 82) plants during the natural flowering period. To minimize developmental variation, only fully expanded flowers at anthesis were selected for analysis. For each genotype, four independent plants were used as biological replicates, and five fully opened flowers were sampled from each individual plant. Floral morphology was examined, and images were captured, using a Leica M205 FA stereomicroscope (Leica Microsystems, Wetzlar, Germany).

Morphometric analysis was conducted using ImageJ software v1.54 [53]. Flower height was defined as the vertical distance from the floral apex to the base of the receptacle, whereas flower width was measured as the maximum horizontal span of the standard petal, following Jin et al. [54]. The flower height-to-width ratio was then calculated as an indicator of floral proportion.

For each biological replicate, the mean value of five flowers was used for statistical analysis. Statistical analyses were performed using SPSS 25. Independent-samples *t*-tests were used to compare flower height, flower width, and flower height-to-width ratio between wild-type and mutant plants. The methods used for normality testing, homogeneity of variance testing, and significance evaluation were the same as those described for seed trait analysis in Section 4.5.

### 4.5. Seed Trait Measurement and Statistical Analysis

To accurately quantify seed morphological variations, seeds were harvested from four independent biological replicates (individual plants) each of the *Gmagl6* quadruple mutants and wild-type (Williams 82). For each plant, five groups of ten mature seeds each were randomly selected as technical replicates. The seeds within each group were aligned linearly without gaps to measure the total length and width using a digital vernier caliper, followed by the measurement of ten-seed weight. The mean, standard deviation, and coefficient of variation (CV) were calculated for the five replicates of each biological individual. Only datasets demonstrating high consistency (CV < 20%) were subjected to further statistical evaluation. Data analysis was conducted using SPSS 25.0. The Shapiro–Wilk test and Levene’s test were employed to verify normality and homogeneity of variance, respectively. Provided that the assumptions of normality and equal variance were met (*p* > 0.05), an independent-samples *t*-test was performed to determine statistical significance between the mutant and wild-type lines.

## 5. Conclusions

In summary, our study identified and characterized four *AGL6* homologs (*GmAGL6a–d*) in the soybean genome. Phylogenetic and structural analyses confirmed that these members belong to the highly conserved MIKC^c^-type MADS-box family and show enriched expression during soybean floral bud differentiation and organ maturation. Functional validation using a CRISPR/Cas9-mediated quadruple mutant population (*Gmagl6*) revealed that, unlike the homeotic transformations observed in monocots such as rice and orchids, *GmAGL6* does not primarily determine floral organ identity in the specialized papilionaceous flowers of soybean. Instead, this gene family may contribute to the regulation of floral proportion and seed size.

These findings suggest that the *AGL6* lineage has undergone substantial functional divergence during angiosperm evolution. In legumes, its role appears to have shifted from ancestral organ identity determination toward the regulation of floral proportion and seed development. Given that seed size is a critical determinant of soybean yield, our results provide a new evolutionary perspective on the developmental mechanisms underlying specialized legume flowers and suggest that *GmAGL6* may represent a potential candidate locus for soybean genetic improvement.

## Figures and Tables

**Table 1:** The gene id of the soybean *AGL6* gene and the gene name used in subsequent studies.

Soybean Gene ID	Gene Name
*Glyma.09G266400*	*GmAGL6a*
*Glyma.18G224300*	*GmAGL6b*
*Glyma.03G019400*	*GmAGL6c*
*Glyma.07G081300*	*GmAGL6d*

**Table 2:** Target sequences and parameters for CRISPR/Cas9-mediated editing of the soybean *GmAGL6* gene family.

Gene Name	Target Name	Target Sequence	Target GC%	Target Domain
*Glyma.09G266400(GmAGL6a)*	Target 1	ACACAGCACTGAGAGCTCTTTGG	50	MADS-box
*Glyma.18G224300(GmAGL6b)*				
*Glyma.09G266400(GmAGL6a)*	Target 2	GTACTTCCGGTATCGATCATAGG	45	MADS-box
*Glyma.18G224300(GmAGL6b)*				
*Glyma.03G019400(GmAGL6c)*	Target 1	CTCATCATTTTCTCAAGCCGTGG	45	MADS-box
*Glyma.07G081300(GmAGL6d)*				
*Glyma.03G019400(GmAGL6c)*	Target 2	AATGGAAGAGCTTCGCAGAAGGG	45	K-box
*Glyma.07G081300(GmAGL6d)*				

## Data Availability

Data are contained within the article and Supplementary Materials.

## Supplementary Materials

The following supporting information can be downloaded at: https://www.mdpi.com/article/10.3390/plants15071070/s1.
