# Supplementary material for: GmAGL6 Genes Regulate Floral Proportion and Seed Size Rather than Keel Petal Identity in Soybean (Glycine max)
Source: Plants (Basel). 2026 Mar 31;15(7):1070. doi: 10.3390/plants15071070 (PMC13074259; doi:10.3390/plants15071070)
Supplement: Supplementary file 1 [file plants-15-01070-s001.zip › plants-4150663-supplementary.pdf]

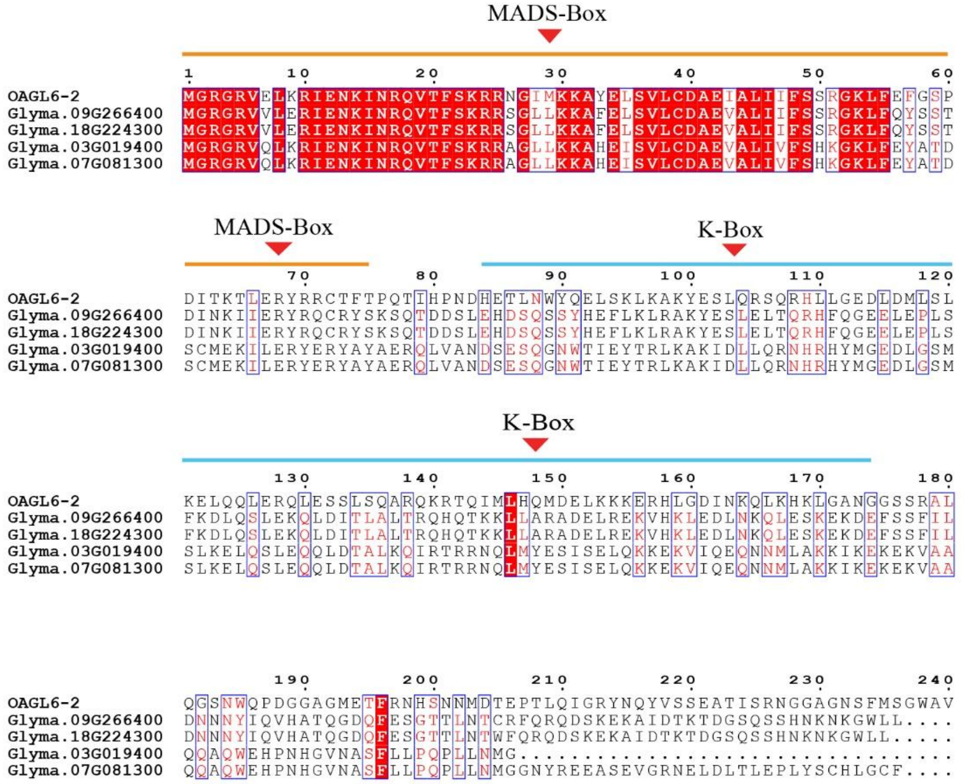

Figure S1. Protein sequence alignment of GmAGL6 homologs in *Glycine max* and *Orchid* OAGL6-2. The conserved MADS-box (orange line) and K-box (blue line) domains are indicated. Red shading and blue boxes highlight identical and physicochemically similar residues, respectively. Numerical coordinates correspond to amino acid positions. Alignment visualization was performed with ESPript 3.0 software.

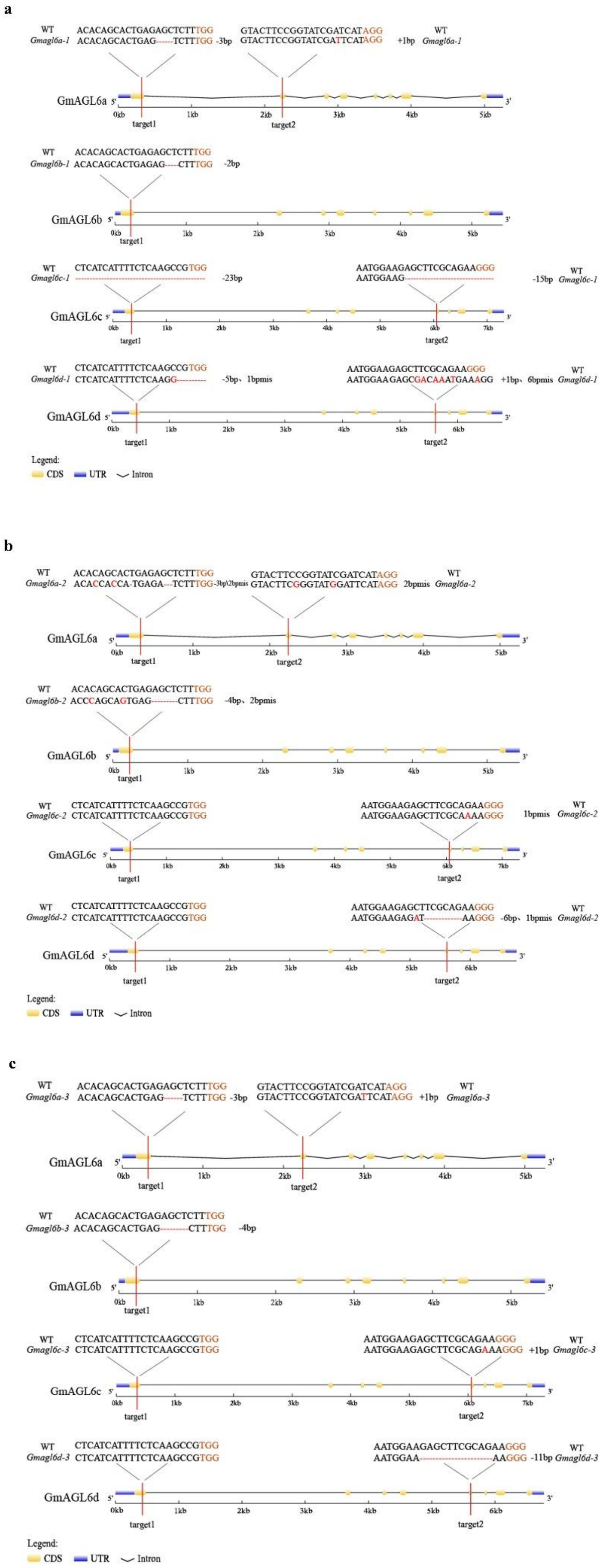

a
WT ACACAGCACTGAGAGCTCTTTGG GTACTTCCGGTATCGATCATAGG WT
Gmagl6a-1 ACACAGCACTGAG------TCTTTGG -3bp GTACTTCCGGTATCGATTCATAGG +1bp Gmagl6a-1
GmAGL6a
0kb 1kb 2kb 3kb 4kb 5kb
target1 target2
WT ACACAGCACTGAGAGCTCTTTGG
Gmagl6b-1 ACACAGCACTGAGAG-----CTTTGG -2bp
GmAGL6b
0kb 1kb 2kb 3kb 4kb 5kb
target1
WT CTCATCATTTTCTCAAGCCGTGG AATGGAAGAGCTTCGCAGAAGGG WT
Gmagl6c-1 -23bp AATGGAAG -15bp Gmagl6c-1
GmAGL6c
0kb 1kb 2kb 3kb 4kb 5kb 6kb 7kb
target1 target2
WT CTCATCATTTTCTCAAGCCGTGG AATGGAAGAGCTTCGCAGAAGGG WT
Gmagl6d-1 CTCATCATTTTCTCAAGG -5bp、1bpmis AATGGAAGAGCGACAAATGAAAGG +1bp、6bpmis Gmagl6d-1
GmAGL6d
0kb 1kb 2kb 3kb 4kb 5kb 6kb
target1 target2
Legend:
CDS UTR Intron
b
WT ACACAGCACTGAGAGCTCTTTGG GTACTTCCGGTATCGATCATAGG WT
Gmagl6a-2 ACACCACCCA-TGAGA---TCTTTGG -3bp\2bpmis GTACTTCGGGTATGGATTCATAGG 2bpmis Gmagl6a-2
GmAGL6a
0kb 1kb 2kb 3kb 4kb 5kb
target1 target2
WT ACACAGCACTGAGAGCTCTTTGG
Gmagl6b-2 ACCCAGCAGTGAG---------CTTTGG -4bp、2bpmis
GmAGL6b
0kb 1kb 2kb 3kb 4kb 5kb
target1
WT CTCATCATTTTCTCAAGCCGTGG AATGGAAGAGCTTCGCAGAAGGG WT
Gmagl6c-2 CTCATCATTTTCTCAAGCCGTGG AATGGAAGAGCTTCGCAAAAGGG 1bpmis Gmagl6c-2
GmAGL6c
0kb 1kb 2kb 3kb 4kb 5kb 6kb 7kb
target1 target2
WT CTCATCATTTTCTCAAGCCGTGG AATGGAAGAGCTTCGCAGAAGGG WT
Gmagl6d-2 CTCATCATTTTCTCAAGCCGTGG AATGGAAGAGAT------------AAGGG -6bp、1bpmis Gmagl6d-2
GmAGL6d
0kb 1kb 2kb 3kb 4kb 5kb 6kb
target1 target2
Legend:
CDS UTR Intron
c
WT ACACAGCACTGAGAGCTCTTTGG GTACTTCCGGTATCGATCATAGG WT
Gmagl6a-3 ACACAGCACTGAG------TCTTTGG -3bp GTACTTCCGGTATCGATTCATAGG +1bp Gmagl6a-3
GmAGL6a
0kb 1kb 2kb 3kb 4kb 5kb
target1 target2
WT ACACAGCACTGAGAGCTCTTTGG
Gmagl6b-3 ACACAGCACTGAG---------CTTTGG -4bp
GmAGL6b
0kb 1kb 2kb 3kb 4kb 5kb
target1
WT CTCATCATTTTCTCAAGCCGTGG AATGGAAGAGCTTCGCAGAAGGG WT
Gmagl6c-3 CTCATCATTTTCTCAAGCCGTGG AATGGAAGAGCTTCGCAGAAAGGG +1bp Gmagl6c-3
GmAGL6c
0kb 1kb 2kb 3kb 4kb 5kb 6kb 7kb
target1 target2
WT CTCATCATTTTCTCAAGCCGTGG AATGGAAGAGCTTCGCAGAAGGG WT
Gmagl6d-3 CTCATCATTTTCTCAAGCCGTGG AATGGAA----------------------AAGGG -11bp Gmagl6d-3
GmAGL6d
0kb 1kb 2kb 3kb 4kb 5kb 6kb
target1 target2
Legend:
CDS UTR Intron

Figure S2. Target site genotyping of *GmAGL6* T0 quadruple mutant lines. (a–c) Schematic representation and sequence analysis of three independent lines: *Gmagl6-1*, *Gmagl6-2*, and *Gmagl6-3*. CDS and UTRs are indicated by yellow and blue boxes, respectively, with introns shown as bent lines. The locations of two CRISPR/Cas9 target sites are indicated by red vertical lines. Mutant sequences are aligned against the wild-type (Williams 82) sequence, with red text or dashes denoting insertions (+), deletions (−), or mismatches (mis).

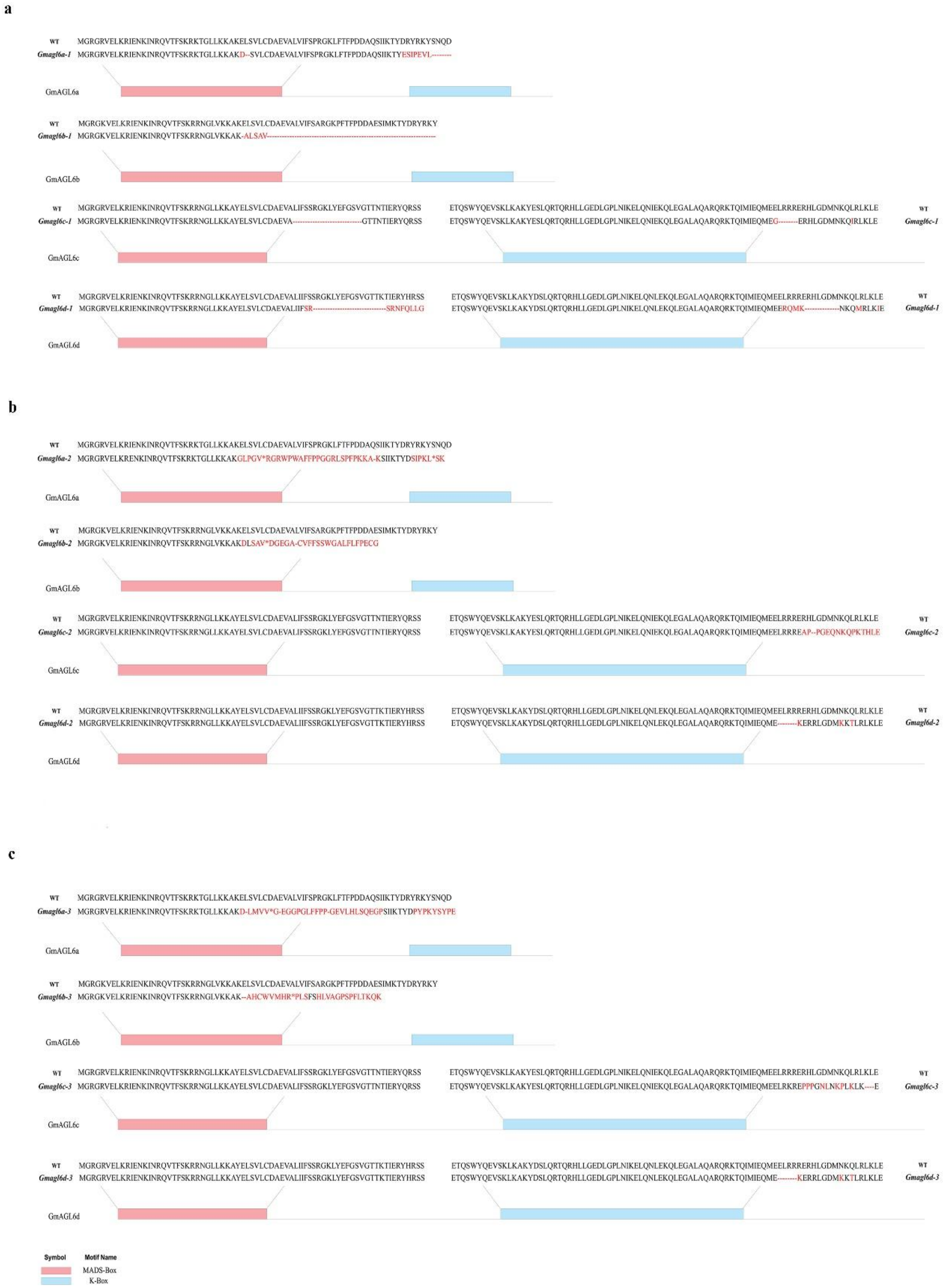
a
b
c
WT
Gmagl6a-1
GmAGL6a
Gmagl6b-1
GmAGL6b
Gmagl6c-1
GmAGL6c
Gmagl6d-1
GmAGL6d
Gmagl6a-2
Gmagl6b-2
Gmagl6c-2
Gmagl6d-2
Gmagl6a-3
Gmagl6b-3
Gmagl6c-3
Gmagl6d-3
Symbol
Motif Name
MADS-Box
K-Box

Figure S3. Protein sequence prediction and alignment of GmAGL6 isoforms in *Gmagl6* quadruple mutant lines. (a–c) Comparative analysis of protein products from three independent lines (*Gmagl6-1* to *Gmagl6-3*). Pink and sky-blue boxes highlight the conserved MADS-box and K-box domains. Red characters indicate frameshift-induced abnormal residues, while asterisks (*) mark premature termination codons (PTCs). Horizontal dashes (---) represent amino acid deletions or C-terminal truncation. WT, wild-type (Williams 82).

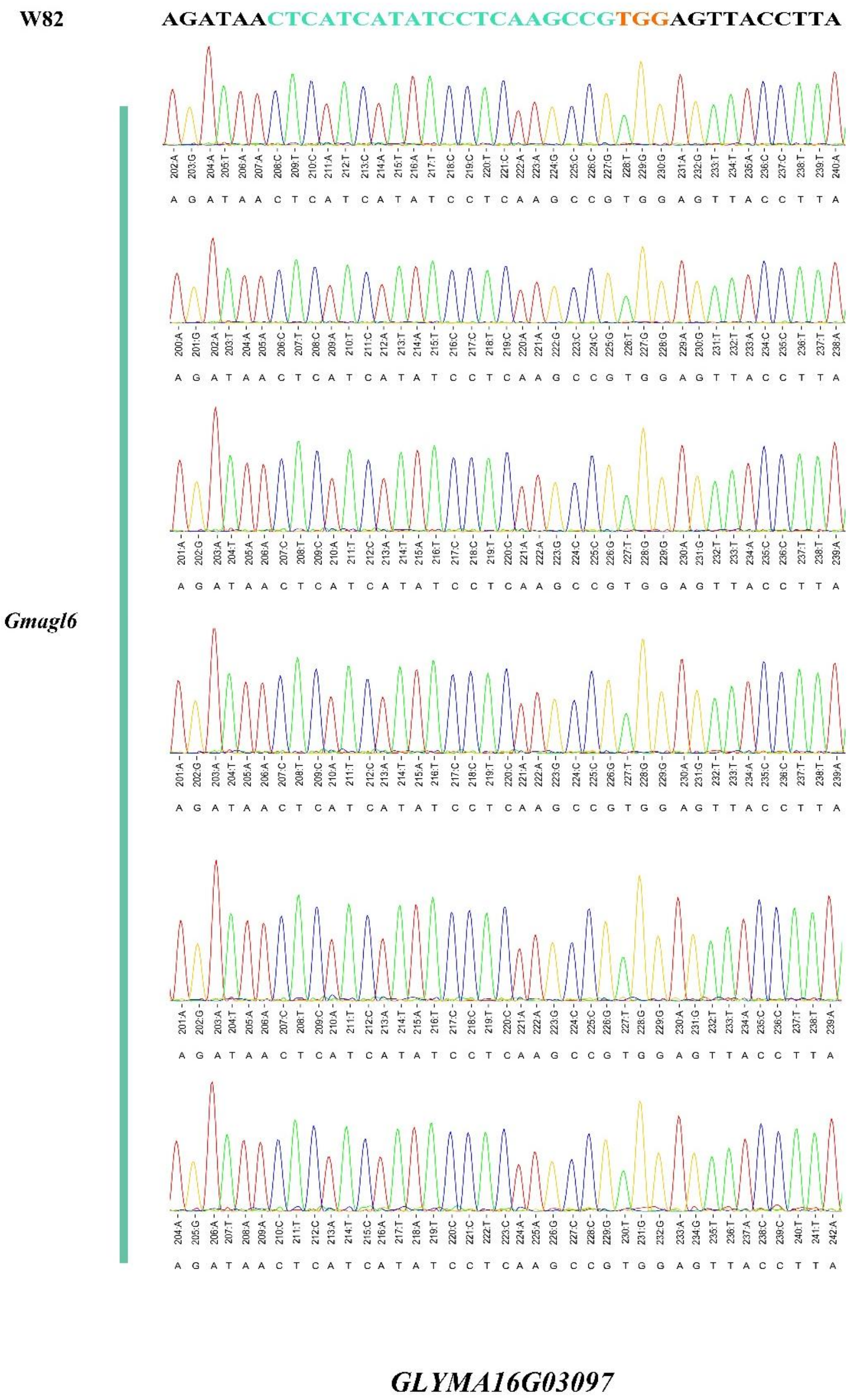

Figure S4. Evaluation of potential off-target effects at the *GLYMA16G03097* locus in *Gmagl6* mutants. The wild-type (Williams 82) reference sequence is shown at the top, with the predicted target sequence and PAM highlighted in green and orange, respectively. Chromatograms from five independent T2 homozygous mutants show clean, single-peak signals matching the WT reference. These results

demonstrate the absence of unintended CRISPR/Cas9-mediated editing at this site, ensuring the genetic reliability of subsequent phenotypic analyses. Alignment and visualization were performed using TBtools.

Table S1. Prediction of potential off-target sites for the *GmAGL6a/b* common Target 1.

Analysis was performed using CRISPR-P 2.0. **Chrom**, chromosome; **Sequence**, potential off-target sequence (red letters: mismatches; green letters: PAM); **Off-score**, value reflecting off-target risk (positive correlation with risk level); **Gene**, associated soybean gene ID; **Region**, genomic localization (CDS, coding sequence; Intron; UTR, untranslated region; Intergenic).

Chrom	Sequence	Off-score	Gene	Region
7	ACACACCACTGAGAGCATTT CGG	0.234	*GLYMA07G02630*	CDS
8	ACACACCACTGAGAGCATTT CGG	0.234	*GLYMA08G23380*	CDS
18	AGAGAAGACTGAGAGCTCTT TGG	0.164		intergenic
7	ACAGAGAACTGAGAGCTCAT AGG	0.107	*GLYMA07G08890*	CDS
20	CCTCAGCACAGAGAGCTGTT TGG	0.069		intergenic
19	GAACGGCTCTGATAGCTCTT TGG	0.067	*GLYMA19G33090*	five_prime_UTR
3	GAACGGCTCTGATAGCTCTT TGG	0.067	*GLYMA03G30190*	five_prime_UTR
6	GCCAAGCACAGAGATCTCTT TGG	0.044	*GLYMA06G40540*	CDS
12	GCCAAGCACAGAGATCTCTT TGG	0.044	*GLYMA12G24910*	CDS
18	GCCAAGCACAGAGATCTCTT TGG	0.044	*GLYMA18G12470*	CDS
4	ACACAGCACTGACAGCTCAT AAG	0.031	*GLYMA04G43640*	CDS
6	ACACAGCACTGACAGCTCAT AAG	0.031	*GLYMA06G48270*	CDS
6	AGACAGCACAGAAAGCACTT TGT	0.005		intergenic
7	ACACAGAACTGAGAGCTCGG TGA	0.002	*GLYMA07G05061*	CDS
20	ACACTGCACTGAGTGCTGCT TTG	0	*GLYMA20G38860*	CDS
10	ACACAGCACTCAGATATCTT TCT	0	*GLYMA10G24391*	intron
16	ACACGGCACAGAGAGCTCTT CAA	0	*GLYMA16G04085*	intron

Table S2. Prediction of potential off-target sites for the *GmAGL6a/b* common Target 2.

Analysis was performed using CRISPR-P 2.0. **Chrom**, chromosome; **Sequence**, potential off-target sequence (red letters: mismatches; green letters: PAM); **Off-score**, value reflecting off-target risk (positive correlation with risk level); **Gene**, associated soybean gene ID; **Region**, genomic localization

(CDS, coding sequence; Intron; UTR, untranslated region; Intergenic)

Chrom	Sequence	Off-score	Gene	Region
15	GTACTTTTGGTACCAATCAA AGG	0.297	*GLYMA15G16960*	CDS
15	GGATTTCTGGCATTGATCAT AGG	0.287		intergenic
12	GTAATTCAGGTATTGACCAT TGG	0.283		intergenic
19	GGTTTTCCGGCATTGATCAT AGG	0.232		intergenic
18	GGATTTCCAGCATTGATCAT AGG	0.211		intergenic
17	GGATTTCCAGCATTGATCAT AGG	0.211		intergenic
16	CTACATCTAGTATCGATCAT TGG	0.201		intergenic
12	GTACTTCCTGCATTCATCAT AGG	0.071	*GLYMA12G03140*	CDS
11	GTACTTCCTGCATTCATCAT AGG	0.071	*GLYMA11G10860*	CDS
2	GTACAACCTGGATCCATCAT AGG	0.025		intergenic
5	GAATTTACTGTATCGATCAC AGG	0.025		intergenic
2	GTACTTGTGGTACCCATCAC AGG	0.008	*GLYMA02G17490*	five_prime_UTR
19	CTACTTCCGGAATTGAGCAT AGT	0.001		intergenic
12	GTACTTTGTGTATGGATCAT AGG	0	*GLYMA12G34480*	CDS
6	GTACTTCTGGTGTGATTCAT AGG	0	*GLYMA06G34225*	intron
19	GTACTTCCTGAATGGATCGA AGG	0	*GLYMA19G32150*	CDS

Table S3. Prediction of potential off-target sites for the *GmAGL6c/d* common Target 1.

Analysis was performed using CRISPR-P 2.0. **Chrom**, chromosome; **Sequence**, potential off-target sequence (red letters: mismatches; green letters: PAM); **Off-score**, value reflecting off-target risk (positive correlation with risk level); **Gene**, associated soybean gene ID; **Region**, genomic localization (CDS, coding sequence; Intron; UTR, untranslated region; Intergenic)

Chrom	Sequence	Off-score	Gene	Region
16	CTCATCATATCCTCAAGCCG TGG	0.619	*GLYMA16G03097*	CDS
14	CTCATCATTTTATCAAATCA TGG	0.402		intergenic
17	CTCATCATCTTCTCCAACCG TGG	0.235	*GLYMA17G08861*	CDS
5	CTCATCATCTTCTCCAACCG TGG	0.235	*GLYMA05G07286*	CDS
8	CTCATCATCTTCTCCAACCG TGG	0.235	*GLYMA08G27670*	CDS
4	CTCATCGTCTTCTCCAGCCG TGG	0.178	*GLYMA04G43640*	CDS
6	CTCATCGTCTTCTCCAGCCG TGG	0.178	*GLYMA06G48270*	CDS
20	CTTATCATCTTCTCCAGCCG TGG	0.173	*GLYMA20G29250*	CDS
10	CTCGTCATCTTCTCCAGCCG TGG	0.157	*GLYMA10G38580*	CDS
13	CTCATTGTTTTCTCTAGCCG TGG	0.131	*GLYMA13G29510*	CDS

20	CTCATCACTTTCATAATCCG TGG	0.127		intergenic
2	CACATCATTTTCACAAGGCG GGG	0.078		intergenic
19	CTCATCATCTTCTCCACCCG TGG	0.059	*GLYMA19G04320*	CDS
12	CTCAGCTTTTTCTCAAGCCG TTG	0.011		intergenic
20	CACAGCTTTTTCTCAAGCCG TTG	0.009		intergenic
9	TTGGTCATTTTCTGAAGCCG TGG	0	*GLYMA09G41570*	five_prime_UTR
19	CTCATAATTTTCTCTTGCCG TAG	0		intergenic

Table S4. Prediction of potential off-target sites for the *GmAGL6c/d* common Target 2.

Analysis was performed using CRISPR-P 2.0. **Chrom**, chromosome; **Sequence**, potential off-target sequence (red letters: mismatches; green letters: PAM); **Off-score**, value reflecting off-target risk (positive correlation with risk level); **Gene**, associated soybean gene ID; **Region**, genomic localization (CDS, coding sequence; Intron; UTR, untranslated region; Intergenic)

Chrom	Sequence	Off-score	Gene	Region
2	TAAGGAAAAGCATCGCAGAA AGG	0.571		intergenic
17	TATTGAACAGCTTCGAAGAA AGG	0.224	*GLYMA17G08432*	CDS
15	TAAGGAAAAGTATCGCAGAA GGG	0.176		intergenic
1	AAAGGATGAGCTTCGCAGTA TGG	0.168	*GLYMA01G36450*	CDS
11	GTCGGAAGAGCTTCCCAGAA GGG	0.085	*GLYMA11G13810*	CDS
9	AAGGGAAGAGCATTGCAGCA GGG	0.061	*GLYMA09G02720*	five_prime_UTR
14	ACTGGAATAGCTTCGCACAA GAG	0.061		intergenic
18	AATGGAAGAGCTTCGTCAAA AGG	0.038	*GLYMA18G45761*	CDS
16	AATGAAAGAGCTACTTAGAA GGG	0.026	*GLYMA16G14385*	CDS
11	ATCGGAAGAGTTTCCCAGAA GGG	0.026	*GLYMA11G13820*	CDS
11	ATCGGAAGAGTTTCCCAGAA GGG	0.026	*GLYMA11G13830*	CDS
13	AATTGAAGAGGTTCTCAAAA GGG	0.009	*GLYMA13G24240*	five_prime_UTR
15	TGTTGAAGAGCTTCTCCGAA GGG	0.007		intergenic
10	AATGGAAGAGCTTTATAGGA GGA	0.006		intergenic
13	AATGGAAGAGCTTTATAGGA GGC	0.002		intergenic
9	CATGGAAGAGCTTGGCCGAA AGG	0	*GLYMA09G08550*	CDS
15	CATGGAAGAGCTTGGCCGAA AGG	0	*GLYMA15G20180*	CDS
17	CGTGGAAGAGTTTGGCAGAA AGG	0	*GLYMA17G05067*	CDS

Table S5. Statistical analysis of floral height in wild-type (Williams82) and *Gmagl6* mutants. For each genotype, floral height was measured using four independent biological replicates for wild-type plants and four independent biological replicates for *Gmagl6* mutant plants. For each biological replicate, five flowers from a single plant were measured, and the average value was used for further analysis. The raw average values for each biological replicate are presented in the "Flower Height" column, with the "Average Value" and "Standard Deviation" calculated accordingly. Normality of data distribution and homogeneity of variance were verified using the Shapiro–Wilk test and the Homogeneity of Variance

Test (Levene's test), respectively. Provided that both assumptions were met ($p > 0.05$), an independent-samples t-test was performed to determine significant differences, with the resulting t-test score and p-value recorded.

Lines	Flower Height(mm)					Avarage Value(mm)	Standard Deviation	CV(%)	Shapiro—Wilk test	Homogeneity of Variance Test	*t*-test score	*p*-value
W82-1	16.52	16.70	17.53	16.64	16.27	16.73	0.48	2.85	0.4	0.3	0.5	0.6
W82-2	16.72	16.57	17.35	15.49	17.35	16.70	0.76	4.56				
W82-3	13.04	16.53	13.57	14.49	14.89	14.50	1.35	9.31				
W82-4	14.25	12.86	13.58	15.57	14.82	14.22	1.05	7.41				
Gmagl6-1	15.71	13.16	15.61	15.58	16.52	15.32	1.27	8.27	0.1			
Gmagl6-2	17.86	14.10	15.11	15.74	15.47	15.65	1.38	8.81				
Gmagl6-3	15.20	15.80	15.65	14.67	15.22	15.31	0.44	2.88				
Gmagl6-4	13.75	13.60	14.53	13.62	12.76	13.65	0.63	4.60				

Table S6. Statistical analysis of floral width in wild-type (Williams82) and *Gmagl6* mutants. For each genotype, floral width was measured using four independent biological replicates for wild-type plants and four independent biological replicates for *Gmagl6* mutant plants. For each biological replicate, five flowers from a single plant were measured, and the average value was used for further analysis. The raw average values for each biological replicate are presented in the "Flower Width" column, with the "Average Value" and "Standard Deviation" calculated accordingly. Normality of data distribution and homogeneity of variance were verified using the Shapiro–Wilk test and the Homogeneity of Variance Test (Levene's test), respectively. Provided that both assumptions were met ($p > 0.05$), an independent-samples t-test was performed to determine significant differences, with the resulting t-test score and p-value recorded.

Lines	Flower Width(mm)					Avarage Value(mm)	Standard Deviation	CV(%)	Shapiro—Wilk test	Homogeneity of Variance Test	*t*-test score	*p*-value
W82-1	12.43	11.20	13.41	11.33	11.49	11.97	0.94	7.82	0.4	0.2	1.4	0.2
W82-2	12.58	11.13	12.87	9.86	12.27	11.74	1.24	10.58				
W82-3	8.67	11.81	10.05	9.98	10.63	10.23	1.14	11.11				
W82-4	7.87	8.92	8.63	11.18	11.00	9.52	1.48	15.60				
Gmagl6-1	10.63	8.39	10.84	10.84	11.14	10.37	1.12	10.84	0.6			
Gmagl6-2	10.64	9.07	10.14	10.99	10.77	10.32	0.76	7.41				
Gmagl6-3	8.57	10.16	11.78	8.93	10.68	10.02	1.31	13.08				
Gmagl6-4	8.27	8.12	8.11	8.65	9.86	8.60	0.74	8.56				

Table S7. Statistical analysis of the floral height-to-width ratio in wild-type (Williams82) and *Gmagl6* mutants. For each genotype, the floral height-to-width ratio was calculated using four independent

biological replicates for wild-type plants and four independent biological replicates for *Gmagl6* mutant plants. For each biological replicate, five flowers from a single plant were measured, and the average floral height and width values were used to calculate the corresponding ratio for further analysis. The raw ratio values for each biological replicate are presented in the “Flower Height/Flower Width” column, with the “Average Value” and “standard deviation” calculated accordingly. Normality of data distribution and homogeneity of variance were verified using the Shapiro–Wilk test and the Homogeneity of Variance Test (Levene’s test), respectively. Provided that both assumptions were met ($p > 0.05$), an independent-samples *t*-test was performed to determine significant differences, with the resulting *t*-test score and *p*-value recorded. “*” indicates a significant difference at $p < 0.05$.

Lines	Flower Height/Flower Width					Avarage Value	Standard Deviation	CV(%)	Shapiro—Wilk test	Homogeneity of Variance Test	*t*-test score	*p*-value
W82-1	1.33	1.49	1.31	1.47	1.42	1.40	0.08	5.78	0.6	0.6	3.4	0.01*
W82-2	1.33	1.49	1.35	1.57	1.41	1.43	0.10	6.99				
W82-3	1.50	1.40	1.35	1.45	1.40	1.42	0.06	4.01				
W82-4	1.81	1.44	1.57	1.39	1.21	1.48	0.22	15.05				
Gmagl6-1	1.48	1.57	1.44	1.44	1.48	1.48	0.05	3.58	0.9			
Gmagl6-2	1.68	1.55	1.49	1.43	1.44	1.52	0.10	6.74				
Gmagl6-3	1.77	1.56	1.33	1.64	1.42	1.54	0.17	11.30				
Gmagl6-4	1.66	1.67	1.79	1.57	1.29	1.60	0.19	11.79				

Table S8. Statistical analysis of the total length of ten seeds in wild-type (Williams82) and *Gmagl6* mutants. For each genotype, five independent Liness (technical replicates, n=5) of ten mature seeds each were randomly selected and aligned linearly without gaps for measurement using a digital vernier caliper. The raw values for each Lines are presented in the "Total Length of Ten Seeds" column, with the "Average Value" and "standard deviation" calculated accordingly. The "CV (%)" (coefficient of variation) was employed as a quality control metric; only datasets demonstrating high consistency (CV < 20%) were subjected to further analysis. Normality of data distribution and homogeneity of variance were verified using the "Shapiro–Wilk test" and the "Homogeneity of Variance Test" (Levene's test), respectively. Provided that both assumptions were met ($p > 0.05$), an independent-samples *t*-test was performed to determine significant differences, with the resulting "*t*-test score" and "*p*-value" recorded. Double asterisks (**) indicate a highly significant difference between Williams 82 and the *Gmagl6* mutant at $p < 0.01$.

Lines	Total Length of Ten Seeds(mm)					Average Value(mm)	Standard Deviation	CV(%)	Shapiro—Wilk test	Homogeneity of Variance Test	*t*-test score	*p*-value
W82-1	73.1	70.96	69.76	74.35	69.49	71.532	2.123	2.969	0.61	0.205	9.408	0.001**
W82-2	69.35	71.15	71.46	70.47	70.44	70.574	0.813	1.152				
W82-3	70.81	70.31	72.04	69.05	70.78	70.598	1.077	1.525				
W82-4	69.26	69.14	68.41	69.44	69.75	69.2	0.498	0.719				
Gmagl6-1	62.05	61.66	60.25	60.04	60.38	60.876	0.912	1.499	0.67			
Gmagl6-2	64.06	63.06	63.03	62.53	63.38	63.212	0.563	0.891				
Gmagl6-3	63.02	60.77	61.27	61.59	61.41	61.612	0.844	1.370				
Gmagl6-4	64.57	64.52	64.41	62.57	63.74	63.962	0.847	1.324				

Table S9. Statistical analysis of the total width of ten seeds in wild-type (Williams82) and *Gmagl6* mutants. For each genotype, five independent Liness (technical replicates, n=5) of ten mature seeds each were randomly selected and aligned linearly without gaps for measurement using a digital vernier caliper. The raw values for each Lines are presented in the "Total Width of Ten Seeds" column, with the "Average Value" and "standard deviation" calculated accordingly. The "CV (%)" (coefficient of variation) was employed as a quality control metric; only datasets demonstrating high consistency (CV < 20%) were subjected to further analysis. Normality of data distribution and homogeneity of variance were verified using the "Shapiro–Wilk test" and the "Homogeneity of Variance Test" (Levene's test), respectively. Provided that both assumptions were met ($p > 0.05$), an independent-samples *t*-test was performed to determine significant differences, with the resulting "*t*-test score" and "*p*-value" recorded. Double asterisks (**) indicate a highly significant difference between Williams 82 and the *Gmagl6* mutant at $p < 0.01$.

Lines	Total Width of Ten Seeds(mm)					Average Value(mm)	Standard Deviation	CV(%)	Shapiro—Wilk test	Homogeneity of Variance Test	*t*-test score	*p*-value
W82-1	64.58	63.26	61.46	64.29	59.6	62.638	2.092	3.340	0.32	0.37	10.069	0.001**
W82-2	61.38	64.15	64.58	62.46	61.82	62.878	1.419	2.257				
W82-3	61.61	62.97	64.44	61.3	60.76	62.216	1.487	2.390				
W82-4	61.7	62.7	62	62.83	61.97	62.24	0.495	0.796				
Gmagl6-1	58.4	57.92	57.41	58.41	57.99	58.026	0.412	0.710	0.83			
Gmagl6-2	58.77	59.2	58.31	59.12	59.97	59.074	0.612	1.036				
Gmagl6-3	57.01	58.63	59.5	57.63	57.98	58.15	0.955	1.643				
Gmagl6-4	57.88	56.1	57.26	57.55	56.62	57.082	0.719	1.259				

Table S10. Statistical analysis of the 100-seed weight in wild-type (Williams 82) and *Gmagl6* mutants. For each genotype, five independent Liness (technical replicates, *n=5*) of ten mature seeds each were randomly selected and weighed using an electronic balance. The 100-seed weight was subsequently estimated based on these ten-seed weight measurements, with the raw data for each Lines presented in the "100-Seed Weight(g)" column. The "Average Value" and "standard deviation" were calculated for each genotype. The "CV (%)" (coefficient of variation) was utilized for quality control; only datasets with high consistency (CV < 20%) were subjected to parametric testing. Normality and homogeneity of variance were verified using the "Shapiro–Wilk test" and the "Homogeneity of Variance Test" (Levene's test), respectively. Provided that the assumptions were met ($p > 0.05$), an independent-samples *t*-test was conducted to determine statistical significance, with the "*t*-test score" and "*p*-value" recorded. Double asterisks (**) indicate a highly significant difference between Williams 82 and the *Gmagl6* mutant at $p < 0.01$.

Lines	100-Seed Weight(g)					Average Value(g)	Standard Deviation	CV(%)	Shapiro—Wilk test	Homogeneity of Variance Test	*t*-test score	*p*-value
W82-1	16.1	16.4	16.8	15	16.6	16.18	0.709	4.379	0.82	0.74	6.11	0.001**
W82-2	15.3	14.2	16.3	16.3	14.7	15.36	0.942	6.135				
W82-3	14.6	15.1	15	14	15.3	14.8	0.515	3.478				
W82-4	16.5	17.1	16.6	16.5	16.9	16.72	0.268	1.605				
Gmagl6-1	10.3	10.6	10.9	10.3	10.8	10.58	0.277	2.623	0.44			
Gmagl6-2	11.6	11.6	11.3	11.3	11.8	11.52	0.217	1.882				
Gmagl6-3	13.2	12.9	11.9	12.4	12.2	12.52	0.526	4.204				
Gmagl6-4	12.9	12.7	12.2	13	12.5	12.66	0.321	2.535				

Table S11. Primers used in this study, including their names, sequences, and specific applications.

Primer Name	Primer Sequence(5′→3′)	Application
Bar-F1	CCATCGTCAACCACTACATCGAGACA	Screening of transgenic positive seedlings of soybean
Bar-R1	CTTCAGCAGGTGGGTGTAGAGCGT	
GmAGL6a-t1-F	GTTCACATGGGGTTGATGCATACG	Detection of the genotypes of each target site in the soybean AGL6 mutant
GmAGL6a-t1-R	AAAGAAGGAGGAGGAGAAAGTAC	
GmAGL6a-t2-F	GACAAAAGAGGTTCAAGTTGGTT	
GmAGL6a-t2-R	GGCTCCATTAACTCAGTAGCTG	
GmAGL6b-t1-F	ACAAGTCCACAACACAGGGAGT	
GmAGL6b-t1-R	ACAAGTCCACAACACAGGGAGT	
GmAGL6b-t2-F1	TCTCCTTTATATCACCCGGCC	
GmAGL6b-t2-R1	TTATTGAAGGGTGGGAGACAG	
GmAGL6b-t2-F2	CCTCTTCACCAACTTCATCTC	
GmAGL6b-t2-R2	GAGCAGAGATGGAATTACACACG	
GmAGL6c-t1-F	CCCTTTTGCACTGAACTTCAC	
GmAGL6c-t1-R	CTATTGGGATTTGCTCCTCAC	

GmAGL6c-t2-F	ATATGGCCTTTAGGCACTTCCC	
GmAGL6c-t2-R	AAGTAAAGCTCAACAAGCTTTCCG	
GmAGL6d-t1-F	CTCTGGAACACACAACACACACA	
GmAGL6d-t1-R	TGCACCTCACAAAAATGGACAC	
GmAGL6d-t2-F	CCGTGCAACAAACATGTCAATATCTAG	
GmAGL6d-t2-R	CCACTATTTCCAGCTTCTGAAGTAG	
GLYMA16G03097-F	TCGACCAGGAAAGGAATCGAAG	To detect the genotype of the potential off-target gene *GLYMA16G03097* and confirm the sequencing results through Sanger sequencing.
GLYMA16G03097-R	CTGACATGACAATCTGACGGTG	
